# Siguiendo la ruta trazada por la Organización Mundial de la Salud: innovación para el control de las enfermedades crónicas no transmisibles

**DOI:** 10.7705/biomedica.7603

**Published:** 2024-05-31

**Authors:** Ricardo A. Peña-Silva, Juan Sebastián Reyes-González

**Affiliations:** 1 Facultad de Medicina, Universidad de los Andes, Bogotá, D. C., Colombia Universidad de los Andes Universidad de los Andes Bogotá, D. C. Colombia; 2 Lown Scholars Program, Department of Global Health and Population, Harvard T. H. Chan School of Public Health, Boston, MA, USA Chan School of Public Health Chan School of Public Health Boston USA

Las enfermedades crónicas no transmisibles se han convertido en una sombra que amenaza la salud y el bienestar de la humanidad. Cada año, millones de vidas se apagan prematuramente debido a la enfermedad cardiovascular, los accidentes cerebrovasculares, el cáncer, la diabetes y las enfermedades respiratorias crónicas. Estas enfermedades, antes asociados con la edad avanzada, hoy afectan a personas de todas las edades, incluso a niños y adolescentes, convirtiéndose en un desafío para la salud pública a nivel mundial. Latinoamérica no es ajena a esta crisis global, enfrentando una creciente carga de enfermedades crónicas no transmisibles que afecta la calidad de vida, la esperanza de vida y el desarrollo socioeconómico de la región. Los sistemas de salud se ven desafiados por la complejidad de estas enfermedades, que requieren tratamientos a largo plazo y un enfoque integral que abarque la prevención, el diagnóstico temprano y la atención continua ^1^^-^^3^.

**Figura 1:**
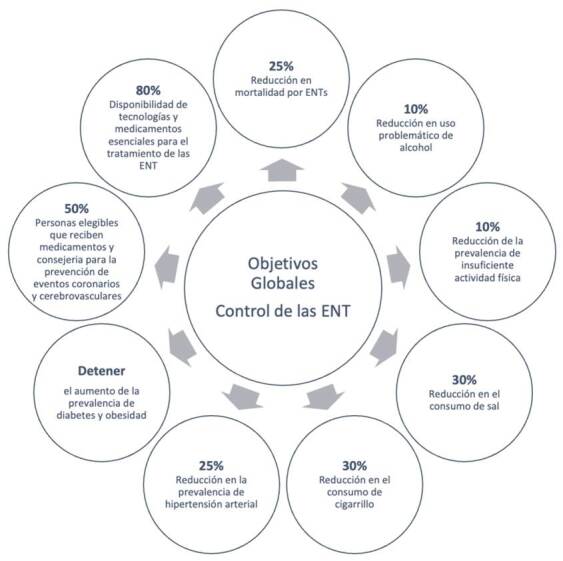
Objetivos globales para el control de las enfermedades crónicas no transmisibles, según la ruta 2023-2030 trazada por la Organización Mundial de la Salud ^2^

La magnitud del desafío exige una respuesta contundente y coordinada. Consciente de la urgencia, la Organización Mundial de la Salud (OMS) ha trazado un camino para la acción global: la Ruta para la Implementación del Plan de Acción Mundial para la Prevención y Control de las Enfermedades Crónicas No Transmisibles, 2023-2030 ^2^. En esta hoja de ruta, que extiende la estrategia original hasta el 2030, se establecen metas ambiciosas para reducir la mortalidad prematura por enfermedades crónicas no transmisibles, disminuir los factores de riesgo -como el tabaquismo, la inactividad física y las dietas poco saludables-, y fortalecer los sistemas de salud para garantizar un acceso equitativo a servicios de calidad (figura 1).

Además, la OMS ha establecido las estrategias de mejor inversión (*best buys*), un conjunto de intervenciones costo-efectivas basadas en evidencia científica, para la prevención y el control de las enfermedades crónicas no transmisibles ^4^. Estas estrategias ofrecen un conjunto de medidas prácticas y accesibles, como aumento de los impuestos al cigarrillo, control de la publicidad relacionada con las bebidas alcohólicas, promoción de dietas saludables y actividad física, y educación en salud. Ambas iniciativas están alineadas con los objetivos de desarrollo sostenible, haciendo evidente la relación entre el bienestar y la salud, el desarrollo social y la sostenibilidad ambiental ^1^^-^^4^.

Este número especial de la revista *Biomédica* ofrece una ventana a la investigación e innovación en el abordaje de las enfermedades crónicas no transmisibles en Latinoamérica y España. Los artículos aquí reunidos nos invitan a explorar diferentes dimensiones de las enfermedades crónicas no transmisibles, desde la investigación básica hasta el manejo clínico y las políticas públicas. Por medio de estudios de casos, análisis epidemiológicos, evaluación de herramientas tecnológicas y revisiones bibliométricas, en este número especial se busca contribuir al conocimiento y a la búsqueda de soluciones para la creciente carga de tales enfermedades en la región.

En el campo de la salud cardiometabólica, es importante considerar la contribución de múltiples factores de riesgo en los desenlaces de salud. La ingestión de grasas y los triglicéridos plasmáticos pueden afectar la concentración de micronutrientes esenciales para el mantenimiento de la homeostasis, como lo muestra el estudio “Circulating zinc levels and cardiometabolic risk-related variables in adults” ^5^.

La diabetes, además, se ha afianzado como un desafío de gran magnitud al afectar a millones de personas en Latinoamérica. Varios artículos de esta edición nos invitan a profundizar en el manejo de esta enfermedad. El estudio “Control glucémico y estudio del metabolismo lipídico y óseo en niños con diabetes de tipo 1”, realizado en España, ilustra cómo el buen control glucémico desde la infancia puede prevenir complicaciones a largo plazo, protegiendo la salud cardiovascular y la ósea ^6^. Este estudio se alinea con las mejores inversiones (*best buys*) que promueven un control glucémico efectivo como una estrategia fundamental para el manejo de la diabetes ^3^.

Por otro lado, los estudios colombianos de investigadores de la Fundación Valle de Lili “Desenlaces clínicos de los pacientes con diabetes e hiperglucemia de estrés que presentaron infección por SARS-CoV-2” ^7^ y “Las crisis hiperglucémicas combinadas en pacientes adultos ya existen en Latinoamérica” ^8^ y “Diabetes mellitus en pacientes con insuficiencia cardiaca y modificación del efecto de los factores de riesgo de mortalidad a corto plazo: un estudio observacional del Registro Colombiano de Falla Cardíaca (RECOLFACA)” ^9^ nos resaltan el impacto negativo de la diabetes y sus complicaciones en pacientes con otras enfermedades, incluyendo las infecciones. El control de la diabetes, por lo tanto, impacta varios niveles la gestión adecuada de las enfermedades crónicas, contribuye a la meta de reducir la mortalidad prematura por enfermedades crónicas no transmisibles y, además, a mejorar la calidad de vida y la esperanza de vida de las personas con diabetes, objetivos que aportan al tercer objetivo del desarrollo sostenible de salud y bienestar.

Las enfermedades renales, a menudo silenciosas pero letales, también requieren de nuestra atención. Los artículos sobre enfermedades quísticas renales “Aspectos genéticos e imagenológicos de la enfermedad quística renal en pediatría” ^10^ y síndrome nefrótico “Evento cerebral isquémico asociado con nefropatía membranosa primaria en un adulto joven: reporte de caso” ^11^, ilustran la heterogeneidad de estas condiciones y la necesidad de un diagnóstico temprano y un manejo adecuado. Las enfermedades renales, a menudo asociadas con otros factores de riesgo como la diabetes y la hipertensión, pueden progresar rápidamente a la insuficiencia renal crónica y requerir diálisis o trasplante. La investigación en este campo, crucial para mejorar el pronóstico de estas enfermedades, se alinea con la meta de fortalecer los sistemas de salud para garantizar un acceso oportuno a servicios de diagnóstico y tratamiento.

Las enfermedades respiratorias, un desafío creciente y que se agrava en entornos con contaminación del aire, también ocupan un lugar destacado en este número especial. La enfermedad pulmonar obstructiva crónica (EPOC), una de las principales causas de muerte a nivel mundial, es el centro de un artículo que nos invita a reflexionar sobre los factores de riesgo, la prevención y el diagnóstico temprano. El estudio “Factores relacionados con la mortalidad en paciente con enfermedad pulmonar obstructiva crónica en población colombiana” revela la asociación entre la exposición al humo de leña, la falla cardiaca y la enfermedad cerebrovascular, y la mortalidad por EPOC ^12^. Este es un llamado a la acción para Latinoamérica, donde el humo de leña y el material particulado amenazan la salud de las comunidades. Estos resultados, además, resaltan la importancia de los equipos interdisciplinarios en la atención de las enfermedades respiratorias crónicas.

La innovación tecnológica también juega un papel crucial en la lucha contra las enfermedades crónicas no transmisibles, como lo demuestra el desarrollo de una aplicación web para el diagnóstico de la EPOC, presentado en “Desarrollo de una aplicación web para evaluar los datos de la espirometría y las variables clínicas para apoyar el diagnóstico de EPOC en atención primaria” ^13^, y el desarrollo de modelos para la evaluación de enfermedades cardíacas “Discriminación de enfermedades cardiacas utilizando patrones cinemáticos codificados con convoluciones 3D en secuencias de cine-RM” ^14^. Estas herramientas demuestran el impacto de la ciencia de datos y los modelos de inteligencia artificial en la medicina contemporánea. Estas técnicas ya han llegado a la región y están siendo adoptadas por hospitales e instituciones académicas. Por medio de la integración de datos clínicos y paraclínicos (como espirometrías o resonancia cardiaca cinemática), se facilita la detección temprana de la EPOC, y establecer el tipo y la extensión del daño de un órgano blanco como el corazón. Estos avances se conectan con la meta de reducir la exposición a factores de riesgo ambientales y de adoptar tecnologías para la gestión de enfermedades crónicas no transmisibles ^2^.

En el campo de la hematología y la oncología, un artículo nos ofrece una mirada a la lucha contra el cáncer en Latinoamérica. “Comparación de las pruebas para el virus del papiloma humano Hybribio-H13 y Hybrid Capture^®^ 2 para la detección de NIC2+ y NIC3+”, en el cual se evalúa la eficacia de una prueba de bajo costo para HPV para detectar lesiones precancerosas de cuello uterino, un paso crucial para la prevención del cáncer ^15^. Este estudio se alinea con las mejores inversiones (*best buys*) que promueven la vacunación contra el HPV y el tamizaje de cáncer de cuello uterino como estrategias costo-efectivas para reducir la incidencia de esta enfermedad ^3^.

Además, otros artículos nos presentan la experiencia de la región en el diagnóstico, seguimiento y manejo de otras neoplasias o trastornos hematológicos. “Uso de romiplostim en trombocitopenia inmunitaria: experiencia en Cuenca (Ecuador)” presenta el nivel de implementación de protocolos y medicamentos para tratar la trombocitopenia ^16^. El caso reportado en “Síndrome de feocromocitoma-paraganglioma de tipo 5 como causa de hipertensión secundaria en una paciente colombiana” muestra la importancia de los estudios genéticos para caracterizar los tipos de tumor y ofrecer un mejor seguimiento a pacientes que presentan tumores productores de hormonas ^17^.

El soporte social y nuestros hábitos de vida modulan la forma en que respondemos ante la enfermedad. “La soledad como predictor de mortalidad en pacientes con cáncer, un estudio de cohorte” nos revela una dimensión a menudo ignorada en el manejo del cáncer: la soledad como factor de riesgo para la mortalidad ^18^. Este estudio pionero en Latinoamérica nos invita a reflexionar sobre la importancia de un enfoque integral que aborde, no solo el tratamiento médico, sino también, el bienestar emocional y social de los pacientes.

Desde el 2022, la *American Heart Association* incluyó el dormir bien como un factor que modula la salud cardiovascular ^19^. En el análisis de los resultados del estudio “Relación de la calidad y la duración del sueño en población colombiana con hipertensión arterial”, no se encontró una relación entre la calidad del sueño y la progresión hacia hipertensión arterial, en población colombiana ^20^. Sin embargo, este abordaje nos recuerda la importancia de incluir en los programas de cuidado de las enfermedades crónicas intervenciones que promuevan hábitos de vida saludable.

El campo de la neurología nos ofrece destellos de esperanza para el futuro. Los estudios sobre la enfermedad de Alzheimer -“Aducanumab: una mirada tras dos años de su aprobación”- ^21^ y la estimulación cerebral no invasiva -“Dominio del conocimiento y tendencias emergentes en estimulación cerebral no invasiva: un análisis bibliométrico a través de CiteSpace” ^22^, nos demuestran que las tecnologías profundas (*deep tech*) ^23^ han llegado a apoyar intervenciones terapéuticas, en entidades que antes considerábamos intratables. Los medicamentos biológicos y las terapias celulares avanzadas están cambiando la historia natural de enfermedades crónicas como el cáncer ^24^. Es crítico que el personal de salud y las personas que están a cargo de las políticas de salud, conozcan más sobre el potencial y las limitaciones de la biotecnología y los dispositivos médicos innovadores, para potenciar la evaluación de estudios de medicamentos biológicos en enfermedades crónicas no transmisibles.

En definitiva, este número especial de la revista *Biomédica* nos ofrece una visión panorámica del desafío que representan las enfermedades crónicas no transmisibles en Latinoamérica. La investigación, la innovación tecnológica, la colaboración multisectorial y la atención centrada en el paciente son los pilares para construir un futuro más saludable y sostenible. Invitamos a los lectores a asumir un rol activo en la lucha contra las enfermedades crónicas no transmisibles, transformando la información en acción.
